# Adaptation, Implementation, and Evaluation of a Public Health Research Methods Training for Youth

**DOI:** 10.1089/heq.2018.0077

**Published:** 2018-11-30

**Authors:** Melody S. Goodman, Ejiro Gbaje, Sallie M. Yassin, Janice Johnson Dias, Keon Gilbert, Vetta Thompson

**Affiliations:** ^1^ Department of Biostatistics, College of Global Public Health, New York University, New York, New York.; ^2^ GrassROOTS Community Foundation, West Orange, New Jersey.; ^3^ Behavioral Science and Health Education, College for Public Health and Social Justice, Saint Louis University, St. Louis, Missouri.; ^4^ Brown School of Social Work, Washington University in St. Louis, St. Louis, Missouri.

**Keywords:** program evaluation, public health, research methods, youth training

## Abstract

**Purpose:** To adapt, implement, and evaluate a public health research methods training program for youth. The Community Research Fellows Training Program is an evidence-based public health research methods training program for adults (18 years and older). The Youth Research Fellows Training (YRFT) is an adaptation of this program for youth.

**Methods:** University faculty facilitate didactic training sessions and experiential small group activities in biweekly sessions conducted as part of an existing 4-week summer camp. Participants were African American girls (*n*=11) ranging from ages 10 to 14 years (most recent grade completed 4th–8th). To evaluate participant knowledge gain and satisfaction pre-tests were administered before each session, and post-test and evaluations were administered after each session. In addition, faculty completed web-based evaluation surveys on their experience teaching in the program.

**Results:** Mean and median post-test scores were higher than pre-test scores for most (6 of the 7) of the training sessions; one session had no difference in scores. Participants rated the sessions well, on average overall session ratings of 4.3–4.8 on a 5-point Likert scale. Faculty rated their experience teaching in the program as excellent or very good and would be willing to teach in the program again (*n*=7; 100%).

**Conclusion:** This pilot implementation of the YRFT program proved highly successful in terms of participant and faculty experience. The program evaluation demonstrates increased knowledge of public health research methods. This program has the potential to prepare youth to engage in public health research as partners not just participants.

## Introduction

The positive youth development approach supports the importance of opportunities for youth engagement.^1^ Engagement allows youth to develop new skills, gain work experience, and develop character attributes such as responsibility, accountability, and integrity.^2^ In addition, evidence suggests that youth who are engaged in community are less likely to be involved in risky behaviors and are more likely to be successful in school,^2^ outcomes important to adult health.

Although there is extensive academic work pertaining to issues affecting youth, it is much less common that youth themselves are included as decision-makers in the research process.^3^ The importance of engaging youth in these spheres of influence was advanced when the 1989 UN Convention on the Rights of the Child explicitly outlined the right for children to actively participate in decision-making processes that are pertinent to them.^4^ Four years later, in 1993, the World Health Organization called for increased youth involvement in setting health initiatives and asserted that effective adolescent health programs depend on the voice of youth.^5^

The benefits of engaging youth in research processes have been discussed extensively in the literature.^6^ They include individual-level benefits to youth of improved self-efficacy, self-empowerment, positive identity formation, and broader community-level benefits of the availability of more effective services and reduced health inequities.^7^ Adolescents are a critical group to include in decision-making because it is at this developmental stage that major health-related choices first begin to happen, and these health behaviors become increasingly difficult to modify into adulthood.^4^ It is imperative that youth, particularly those of minority populations who carry a disproportionate burden of negative health outcomes, are engaged as active informants in research to most successfully develop solutions to health problems that only they can accurately contextualize.

Although the prevalence of Community-Based Participatory Research (CBPR) involving youth is thought to be increasing, some researchers have noted that it can be hard to determine to what degree youth are truly partnering with researchers as CBPR can be defined and interpreted differently among researchers.^3^ Although the general tenets of CBPR remain the same regardless of the age of the population involved, there do exist some differences when partnering with youth and respective practices that are common in most effective youth CBPR programs. In a review by the *Harvard Family Research Group* (2002), five key elements were identified as being essential for successful youth-involved research and evaluation projects: (1) organizational and community readiness, (2) adequate support and training for involved youth, (3) adequate support and training for adult staff, (4) selecting the right team, and (5) sustaining youth involvement. An important component in the success of programs is the attention given to coaching the adults to act as facilitators, not as authoritative figures, to give youth the driving voice of the project.^5,8^

Many studies identified motivating factors that were important in sustaining the active engagement of the youth partners, including the opportunity to change the perspectives of adults and for research to be a vehicle for change.^9^ The significance of representation in terms of both underserved populations as well as researchers reflecting the youth they are partnering with is a repeated theme discussed throughout the literature base.^10,11^ Although the importance of engaging marginalized populations in researching solutions to the problems they disproportionately face is widely accepted in the literature, a study by Galletta and Jones discusses how critical it is that there is representation of these communities among the adult researchers as well.^12^

The Youth Research Fellows Training (YRFT) program is a public health research methods training program for youth adapted from the Community Research Fellows Training (CRFT) program. The CRFT program is an evidence-based training program to increase research literacy among adults (≥18 years old) based on a standard Masters in Public Health (MPH) curriculum. The goal of CRFT is to increase the role of minority and medically underserved communities in the research enterprise by developing the foundational infrastructure for community-academic partnerships through community research capacity building. CRFT has been implemented and evaluated in St. Louis, MO (four cohorts), Jackson, MS (two cohorts), and Hattiesburg, MS (one cohort); details about program implementation and evaluation have been discussed elsewhere.^13–18^

The goal of the YRFT program is to provide training on public health research and use of data for youth activist interested in conducting social action projects. Trainees gain an understanding of the research process, research methods, and sources of data that can be used to explain, document, and evaluate progress on social justice issues in their communities. In this study, we discuss the implementation and evaluation of the YRFT pilot.

## Methods

### Program description

The principal investigator of the YRFT program collaborated with the president of the GrassROOTS Community Foundation (GCF), to pilot YRFT as a component of the GFC 2018 Super Camp in Essex County, New Jersey. The YRFT piloted in July–August 2018 was adapted for youth from the CRFT, an evidence-based public health research methods training program for adults.^13–18^ The 4-week program met twice each week for 90 min on Tuesdays and 75 min on Thursdays. In addition to participating in the on-site training sessions, participants were given four homework assignments (windshield survey, playground audit, Photovoice draft, and Photovoice final). At the first session, participants received supplies needed to participate in the training, including a backpack, binder, notepad, and pens. Handouts with session presentation and activities were provided at each session. Supplies needed for breakout activities (e.g., crayons, markers, color pencils, and pens) were provided when needed.

Session topics and learning objectives are presented in Table 1; session topics include the following: Introduction to Public Health Research, Social Determinants of Health, Research Methods, Health Disparities, Qualitative Methods: Understanding Community Health, Quantitative Research Methods, and Using Research and Data for Social Justice. At the last session, fellows presented their final Photovoice homework assignment and received their certificate of completion. Training sessions had a similar format to CRFT with each facilitated by a different instructor using PowerPoint for the didactic training component followed by small group breakout activity, and concluding with a report back to the larger group. Breakout session activities were region specific and tailored to the participant demographics.

**Table 1.** **Session Topics and Learning Objectives**

Topics	Learning objectives
Session 1A	
Introduction to Public Health Research	Define public health
	Describe the research process
	Explain why research is important
Session 1B	
Social Determinants of Health	Define the social determinants of health
	Describe the fundamental causes of disease/illness
	Identify examples of effective local, regional, and national strategies for improving systems and policies that affect the social determinants of health
	Discuss how where you live affects your health
Session 2A	
Research Methods	Develop data collection strategies to address a research problem
	Determine appropriate sampling methods to address a research question
	Compare and contrast primary data and secondary data
Session 2B	
Health Disparities	Describe health disparities
	Explain how inequities in neighborhood conditions, education, income and wealth, and sociopolitical climate affect health outcomes and health disparities
	Define the generations of health disparities research
Session 3A	
Qualitative Methods: Understanding Community Health	Utilize different types of qualitative data and data sources to describe communities
	Compare and contrast quantitative and qualitative data
	Discuss qualitative data collection methods (e.g., focus groups, interviews, and Photovoice)
Session 3B	
Quantitative Research Methods	Utilize different types of quantitative data and data sources to describe communities
	Develop survey instruments for data collection
	Draw appropriate conclusions from data
	Draw appropriate conclusions from tables, graphs, and figures
Session 4A	
Using Research and Data for Social Justice	Identify major health disparities in the region, including those by gender, race/ethnicity, geographic location, and socioeconomic status
	Identify contributing factors that impact the health of a community
	Identify and develop relevant well-framed community organizing strategies
Session 4B	
Photovoice Presentation and Certificate of Completion	Fellows present Photovoice homework assignment
	Fellows receive certificate of completion

### YRFT faculty

The first seven sessions were led by faculty from New York University College of Global Public Health (*n*=3), Brown School of Social Work at Washington University in St. Louis (*n*=3) and the College for Public Health and Social Justice at Saint Louis University (*n*=1). The majority (*n*=5; 71%) of the faculty were African American, women, and had previously taught in the CRFT program for adults. The majority of the faculty are in public health (*n*=6; 86%) and cover a range of health disciplines, including behavioral science, health education, biostatistics, epidemiology, social science, clinical psychology, and social work.

### Program evaluation

We conducted a comprehensive evaluation of the YRFT program to assess participant knowledge and satisfaction and faculty experiences. The YRFT evaluation team consisted of a principal investigator and three research assistants (two undergraduate and one MPH student). The YRFT evaluation was approved by New York University Institutional Review Board (NYU IRB)/University Committee on Activities Involving Human Subjects, Office of Research Compliance. Both parental consent and participant assent were obtained for the program evaluation. Statistical analysis was conducted using SAS 9.4 (Cary, NC); statistical significance is assessed as *p*<0.05.

### Assessment of participant knowledge

The research fellows took pre-tests before each training session and post-tests after each of the seven training sessions. The tests were written by the YRFT evaluation team and approved by the faculty member teaching the session. Each of the tests consisted of 10 multiple-choice questions. The pre- and post-tests had the same questions but in differing order and were linked using participant ID numbers. The questions assessed the learning objectives that were intended to be covered by the session's instructor. The tests were graded by YRFT research assistants using an answer key developed for the pre- and post-tests. Given the small sample size and paired pre-/post-test data, the nonparametric Wilcoxon signed-rank test was used to test median differences between the pre- and post-test scores for each session.

### Participant evaluation

At the end of each session (1–7), participants completed a session evaluation. At the beginning of the last session, participants completed a program evaluation. Session evaluations were anonymous and consisted of five multiple-choice Likert response questions with a rating scale of 1 “strongly disagree” to 5 “strongly agree,” one multiple-choice Likert response questions with a rating scale of 1 “poor” to 5 “excellent,” and four open-ended questions. Closed-ended questions assessed whether the session learning objectives were met, information from the session was helpful, concepts presented were understood, the teacher was organized, the teacher was knowledgeable, and an overall rating for the session. Open-ended questions assessed the three most important things learned during the session, what was liked most about the session, what was not liked about the session, and any additional comments or suggestions.

### Faculty evaluation

At the completion of the program, faculty were sent an email to participate in a web-based evaluation survey using Qualtrics survey software. Two weeks after the initial invitation, an automated system reminder was sent to faculty with incomplete surveys. The faculty evaluation survey consisted of 11 closed-ended and 3 open-ended questions. Eight of the closed-ended items were asked on 5-point Likert scale from “strongly disagree” to “strongly agree” and assessed fellows' preparation, engagement, and participation in the training session. Faculty were also asked if they would teach in the YRFT program again (yes/no), the appropriate grade level for the youth program (middle school/high school/undergraduate), and to rate their overall experience teaching in the program (poor/fair/good/very good/excellent). Open-ended questions assessed structural/format changes for the program, memorable moments during teaching, and additional suggestions/comments for the program.

## Results

### Participant characteristics

All YRFT fellows (*n*=11) were participants of the GCF 2018 New Jersey Super Camp; African American girls ranging from ages 10 to 14 years (most recent grade completed 4th–8th). The YRFT program was imbedded into the Super Camp schedule as a result the majority (*n*=10; 91%) of participants had perfect attendance; one participant missed one session and one participant left one session early. All participants completed three homework assignments (windshield survey, Photovoice draft, and Photovoice final); one participant did not complete the playground audit assignment (Table 2).

**Table 2.** **Demographic Characteristics, Attendance, and Homework Completion of Youth Research Fellows Training Participants (*n*=11)**

Characteristic	*n*	%
Age (years)		
10	6	54.5
11	1	9.1
12	1	9.1
13	2	18.2
14	1	9.1
Last grade of school completed		
4th grade	2	18.2
5th grade	5	45.5
6th grade	1	9.1
8th grade	3	27.3
Number sessions attended		
8	10	90.9
7	1	9.1
Homework completed		
Windshield survey	11	100.0
Playground audit	10	90.9
Photovoice draft	11	100.0
Photovoice final	11	100.0

### Pre- and post-test analysis

When comparing pre-test and post-test scores, mean and median post-test scores were higher than pre-test scores for six of the seven of the training sessions; one session had no difference in scores (2B: health disparities), and this session had the highest mean (77%) and median (80%) pre-test score. Based on the Wilcoxon Signed-Ranked test, the increases in scores were statistically significant in four sessions (Table 3): session 1A: Introduction to Public Health Research (*p*=0.016), session 1B: Social Determinants of Health (*p*=0.004), session 2A: Research Methods (*p*=0.047), and session 4A: Using Research and Data for Social Justice (*p*=0.023).

**Table 3.** **Analysis of Youth Research Fellows Training Pre- and Post-test Scores by Session**

		Pre-test percent			Post-test percent			Score difference			Wilcoxon signed-rank test	
Session	*n*	Mean	Median	SD	Mean	Median	SD	Mean	Median	SD	Statistic	*p*
1A. Introduction to Public Health Research	11	68.3	75.0	25.2	82.7	90.0	20.5	15.5	10.0	18.1	14.0	0.016^a^
1B. Social Determinants of Health	11	65.0	70.0	12.4	79.1	90.0	17.6	15.5	20.0	9.3	22.5	0.004^a^
2A. Research Methods	10^b^	59.0	65.0	19.1	70.0	70.0	19.4	11.0	15.0	12.9	12.5	0.047^a^
2B. Health Disparities	11	77.3	80.0	11.0	77.3	80.0	19.5	0.0	0.0	12.6	-0.5	1.000
3A. Qualitative Methods: Understanding Community Health	11	42.7	40.0	23.3	56.4	60.0	21.6	13.6	20.0	22.9	14.5	0.102
3B. Quantitative Research Methods	10	62.0	65.0	14.8	65.0	70.0	23.2	3.0	10.0	20.0	7.0	0.219
4A. Using Research and Data for Social Justice	11	70.0	70.0	14.1	80.9	80.0	16.4	10.9	10.0	11.4	19.5	0.023^a^

^a^ *p*<0.05.

^b^ One participant left early and did not complete the post-test; excluded from analysis.

### Participant session evaluation

Participants rated the sessions positively, with means for each question across sessions ranging from 4.0 to 4.9 on a 5-point Likert scale (Table 4). For overall evaluation of the session, mean scores ranged from 4.3 (research methods; health disparities) to 4.8 (quantitative research methods). In addressing what they did not like about the program, several participants mentioned lack of time, feeling rushed, and instructors moving through the material too quickly. They really enjoyed the use of videos and the breakout group activities.

**Table 4.** **Summary of Participant Evaluations**

	1. The session learning objectives were met			2. The information learned in this session was helpful			3. I understand the concepts presented in this session			4. The teacher(s) were well organized			5. The teacher(s) seemed knowledgeable about the subject			6. Overall, how would you rate this session?		
	Mean	Median	SD	Mean	Median	SD	Mean	Median	SD	Mean	Median	SD	Mean	Median	SD	Mean	Medan	SD
1A Introduction to Public Health Research	4.2	4.0	0.6	4.5	5.0	0.8	4.0	4.0	0.7	4.6	5.0	0.6	4.9	5.0	0.3	4.5	4.5	0.5
1B Social Determinants of Health	4.5	4.5	0.5	4.5	5.0	0.6	4.3	4.5	0.8	4.4	5.0	0.8	4.8	5.0	0.4	4.6	5.0	0.5
2A Research Methods	4.3	4.0	0.7	4.2	4.0	0.6	4.2	4.0	0.6	4.6	5.0	0.7	4.8	5.0	0.4	4.3	4.0	0.7
2B Health Disparities	4.5	5.0	0.6	4.5	5.0	0.8	4.3	4.5	0.7	4.4	4.0	0.6	4.5	5.0	0.4	4.3	4.0	0.7
3A Qualitative Methods: Understanding Community Health	4.7	5.0	0.6	4.6	5.0	0.6	4.5	4.3	0.5	4.8	5.0	0.4	4.8	5.0	0.4	4.7	5.0	0.5
3B Quantitative Research Methods	4.8	5.0	0.4	4.5	5.0	0.8	4.6	5.0	0.6	4.8	5.0	0.4	4.8	5.0	0.4	4.8	5.0	0.4
4A Using Research and Data for Social Justice	4.8	5.0	0.4	4.5	5.0	0.5	4.5	5.0	0.5	4.7	5.0	0.4	4.8	5.0	0.4	4.6	5.0	0.5
4B Program Overall	4.5	4.0	0.5	4.6	5.0	0.6	4.5	5.0	0.7	4.6	5.0	0.5	4.8	5.0	0.4	4.7	5.0	0.4

Questions 1–5 based on a 5-point Likert scale with 1 being strongly disagree and 5 being strongly agree.

Question 6 based on a 5-point Likert scale with 1 being poor and 5 being excellent.

SD, standard deviation.

### Faculty evaluation

All participating faculty completed the evaluation survey (*n*=7). Faculty rated their experience teaching in the program as excellent (*n*=5; 71%) or very good (*n*=2; 29%) and would be willing to teach in the program again (*n*=7; 100%). The mean response for the seven Likert response questions related to fellows participation and engagement ranged from 4.4 (fellows took notes during the session) to 5.0 (fellows provided insightful comments; fellows asked constructive questions). All faculty stated that the fellows were engaged in the breakout activity during their session and the program would be good for middle school, high school, and undergraduates. Faculty commented on how this program has the potential to enhance the public health pipeline and demonstrates the importance of exposing youth to public health. When asked what they would change about the program, location (temperature and noise level) and session length were two common themes across faculty responses (data not shown).

## Discussion

The program was adapted from CRFT by reducing the number of sessions and the time allotted for each session. The majority of session topics are directly from the CRFT program (e.g., public health research, research methods, health disparities, social determinants of health, and quantitative research methods); the qualitative methods session was adapted to qualitative methods: understanding community health and one new session was added using research and data for social justice. There were 15 training sessions in CRFT and 7 for YRFT. CRFT sessions were once a week for 3 hours and YRFT sessions were twice a week for 90 and 75 minutes for a total of 2 hours and 45 minutes per week. However, many of the YRFT sessions started early allowing for additional time. We believe that 2 hours per session would be adequate for the instructor's presentation and the small breakout group activity. The four YRFT homework assignments were adapted for youth from the six CRFT homework assignments. While CRFT participants had 2 weeks to complete each assignment, YRFT participants were given assignments the week before they are due. Each assignment was modified to be shorter and appropriate for youth (e.g., playground audit instead of grocery store audit).^14^

Adapting a graduate-level public health curriculum for youth was a major challenge for YRFT faculty. In addition to the reduced session time allowed to cover to the material. Key to the success of the adaption was limiting the number of PowerPoint slides to 15–20, using YouTube videos to reinforce key concepts, and adapting the breakout activities to feel like games. The training format allowed us to routinize the participants enabling completion of session tasks in the time allotted. Similar to CRFT each session was facilitated by a different faculty member exposing participants to university faculty all of who have the same gender and/or race of the participants. Similar to the CRFT faculty, the YRFT is very diverse in terms of gender and race providing great exposure for youth to see professors who look like them.

This pilot implementation and evaluation study has provided evidence that the CRFT program curriculum can be tailored and adapted to other populations. However, the results should be considered in light of several study limitations that preclude generalizability. The sample size is small (*n*=11), which is adequate for pilot implementation and nonparametric statistical tests are used to examine differences in pre-test and post-test scores. The sample is all African American girls as it was implemented as part of the GCF Super Camp for African American girls. This sample is unique as both the girls and parents must complete an application and interview to be accepted into the camp. The camp focuses on social justice issues providing a unique sample of girls that were actively engaged in the training program. Most of the YRFT faculty were from the CRFT faculty having previously taught for multiple CRFT cohorts and were used to the format of the training.

Despite these limitations, this pilot implementation of the YRFT program proved highly successful in terms of participant satisfaction. The fellows were excited to take a college style course taught by college faculty and receive certificates of completion with a college logo. The participants created a video about their participation in the program; two participants developed qualitative interview questions and interviewed other participants about their thoughts and experience in the program. This unanticipated outcome demonstrated key skills learned in the program about collecting data and using data to tell a story.

### Health equity implications

The results of the pilot program has strong implications for future implementation among larger cohorts of youth, the potential for integration into middle and high school curriculum, tailoring for other racial/ethnic minority subgroups, and broader dissemination in other geographic locations. This work makes a significant contribution to enhancing the infrastructure for youth-academic partnerships in public health increasing the potential for studies that address issues of importance to youth and can lead to the reduction of health disparities.

## Abbreviations Used

CBPR: Community-Based Participatory Research
CRFT: Community Research Fellows Training
GCF: GrassROOTS Community Foundation
MPH: Masters in Public Health
SD: standard deviation
YRFT: Youth Research Fellows Training

## References

[1] Sherrod LR, Torney-Purta J, Flanagan CA Handbook of Research on Civic Engagement in Youth. Hoboken, NJ: John Wiley & Sons, 2010

[2] Center for the Study of Social Policy. Results-Based Public Policy Strategies for Promoting Youth Civic Engagement. Available at: https://www.cssp.org/policy/papers/Promoting-Youth-Civic-Engagement.pdf Accessed September 8, 2018

[3] Jacquez F, Vaughn LM, Wagner E Youth as partners, participants or passive recipients: a review of children and adolescents in community-based participatory research (CBPR). Am J Community Psychol. 2013;51:176–189 22718087 10.1007/s10464-012-9533-7

[4] MacDonald J-AM, Gagnon AJ, Mitchell C, et al. Include them and they will tell you: learnings from a participatory process with youth. Qual Health Res. 2011;21:1127–1135 21508252 10.1177/1049732311405799

[5] Suleiman AB, Soleimanpour S, London J Youth action for health through youth-led research. J Community Pract. 2006;14:125–145

[6] Jardine CG, James A Youth researching youth: benefits, limitations and ethical considerations within a participatory research process. Int J Circumpolar Health. 2012;71:18415 10.3402/ijch.v71i0.18415 PMC3417519 22584512

[7] Ozer EJ, Douglas L The impact of participatory research on urban teens: an experimental evaluation. Am J Community Psychol. 2013;51:66–75 22875686 10.1007/s10464-012-9546-2

[8] Horsch K, Little PMD, Smith JC, et al. Youth involvement in evaluation and research. Harvard Family Research Project: Issues and Opportunities in Out-of-School Time Evaluation Briefs. No. 1. Available at: http://www.hfrp.org/publications-resources/browse-our-publications/youth-involvement-in-evaluation-research Accessed 98, 2018

[9] Mmari K, Blum R, Sonenstein F, et al. Adolescents' perceptions of health from disadvantaged urban communities: findings from the WAVE study. Soc Sci Med. 2014;104:124–132 24581070 10.1016/j.socscimed.2013.12.012

[10] Hussain-Gambles M, Atkin K, Leese B Why ethnic minority groups are under-represented in clinical trials: a review of the literature. Health Soc Care Community. 2004;12:382–388 15373816 10.1111/j.1365-2524.2004.00507.x

[11] Ollner A A Guide to the Literature on Participatory Research with Youth Student The Assets Coming Together for Youth Project, 2010 www.yorku.ca/act Accessed 913, 2018

[12] Galletta A, Jones V “Why are you doing this?” Questions on purpose, structure, and outcomes in participatory action research engaging youth and teacher candidates. Educ Stud. 2010;46:337–357

[13] Komaie G, Ekenga CC, Thompson VLS, et al. Increasing community research capacity to address health disparities: a qualitative program evaluation of the community research fellows training program. J Empir Res Hum Res Ethics. 2017;12:55–66 28220721 10.1177/1556264616687639 PMC5321619

[14] Komaie G, Gilbert KL, Arroyo C, et al. Photovoice as a pedagogical tool to increase research literacy among community members. Pedagog Heal Promot. 2017;4:2373379917715652

[15] Coats JV, Stafford JD, Sanders Thompson V, et al. Increasing research literacy: the community research fellows training program. J Empir Res Hum Res Ethics. 2015;10:3–12 25742661 10.1177/1556264614561959 PMC4605821

[16] Fastring D, Mayfield-Johnson S, Funchess T, et al. Increasing research capacity in underserved communities: formative and summative evaluation of the Mississippi community research fellows training program (cohort 1). Front Public Heal. 2018;6:21 10.3389/fpubh.2018.00021 PMC5811515 29479526

[17] McGowan LD, Stafford JD, Thompson VL, et al. Quantitative evaluation of the community research fellows training program. Front Public Heal. 2015;3:179 10.3389/fpubh.2015.00179 PMC4504145 26236703

[18] Goodman MS, Thompson VS, eds. Public Health Research Methods for Partnerships and Practice. Abingdon, Oxon: Routledge/Taylor & Francis Group, 2018

